# Audit of Group and Save Sample Rejection in Fractured Neck of Femur Patients at a United Kingdom District Hospital

**DOI:** 10.7759/cureus.102811

**Published:** 2026-02-02

**Authors:** Bhavna Shah, Tarani Sai Prasanth Grandhi, Zara Syeda, Esther Idowu, Nanda Chetty, Amit Sharma

**Affiliations:** 1 Trauma and Orthopaedics, Luton and Dunstable Hospital, Luton, GBR

**Keywords:** blood transfusion safety, clinical audit, emergency department, group and save, sample rejection, trauma and orthopaedics

## Abstract

Background: Accurate preoperative blood grouping and antibody screening are critical for trauma and orthopaedic (T&O) surgery. Group and Save (G&S) samples are frequently rejected due to labelling discrepancies, incomplete documentation, or sample quality issues. Such rejections delay surgery, increase patient discomfort from repeated venepuncture, and generate avoidable financial and operational burdens. This audit assessed the frequency, underlying causes, and financial implications of rejected GS samples for T&O patients in the Accident and Emergency (A&E) department and developed targeted recommendations to minimise error rates.

Methods: A retrospective clinical audit was conducted at the Luton and Dunstable Hospital NHS Foundation Trust, United Kingdom, encompassing all patients admitted with neck of femur (NOF) fractures between September 2024 and March 2025. Laboratory information system data and request forms were reviewed to identify rejected samples, categorise causes of rejection, and determine the staff group responsible for collection. Cost estimates were calculated using the hospital's local pathology finance reports. A follow-up re-audit was conducted after implementing the initial audit's recommendations, from July to October 2025.

Results: Of 171 G&S samples, 64 (37.4%) were rejected. The majority (62.5%, n=40) were taken by A&E staff. Predominant causes were absent signatures, missing date/time, mismatched identifiers, and sample integrity issues (haemolysis/underfilling). Approximately 20% (n=13) of affected patients required ≥2 repeat samples. The direct cost per rejected sample ranged from £12 to £80. After interventions, a re-audit of 84 samples showed the rejection rate fell to 16.6% (n=14).

Conclusions: Sample rejection in A&E is a preventable source of perioperative inefficiency. Most errors stemmed from documentation/labelling lapses. Implementing standardised bedside labelling, electronic order validation, and mandatory competency refreshers supported by a G&S checklist substantially reduced rejection rates. These measures optimise workflow, reduce costs, and enhance patient safety.

## Introduction

Accurate preoperative blood grouping and antibody screening are essential components of surgical safety and transfusion preparedness, particularly in trauma and orthopaedic (T&O) patients who frequently require operative intervention under urgent circumstances. Robust protocols for ABO verification are a cornerstone of this process, helping to mitigate the risk of critical transfusion errors [1].

Previous studies have reported that preanalytical errors account for up to 60-70% of total laboratory mistakes, with patient identification and labelling discrepancies being the most common causes [2]. Within Accident and Emergency (A&E) departments, Group and Save (G&S) or crossmatch samples are commonly collected at the time of patient admission. These samples are later used to confirm blood type and screen for antibodies before surgery. However, errors in sample labelling, incomplete documentation, and compromised sample quality remain recurring problems that lead to sample rejection in hospital laboratories [2,3]. Such errors are a recognized source of 'wrong blood in tube' incidents, a serious patient safety concern documented in national hemovigilance reports [4].

Sample rejection can have significant clinical and operational consequences. Rejected G&S samples necessitate repeat venepuncture, delaying surgical scheduling and increasing patient discomfort and anxiety. In acute trauma cases such as neck of femur (NOF) fractures, where early surgery is linked to reduced morbidity and mortality, any delay in preoperative work-up may directly affect clinical outcomes [5,6]. Additionally, each rejection generates avoidable expenditure through wasted materials, additional laboratory processing, and staff time [7]. This inefficiency also has an environmental impact: repeated sample collection, processing, and disposal consume additional single-use plastics (e.g., tubes, needles, packaging) and energy for transportation, storage, and laboratory analysis, thereby increasing the carbon footprint of clinical care [8].

Within the United Kingdom's National Health Service (NHS), several institutions have undertaken local quality improvement initiatives to address this issue; however, the persistence of sample rejections in emergency settings suggests that procedural lapses and training deficits remain. At the Luton and Dunstable Hospital, internal laboratory reports indicated a high incidence of rejected A&E and G&S samples for trauma patients, prompting the need for a structured audit to identify root causes and implement corrective strategies.

This clinical audit aimed to determine the frequency and reasons for rejection of G&S samples collected in A&E for patients admitted with NOF fractures, assess the financial impact of these rejections, and develop and evaluate evidence-based recommendations to minimise future occurrence and improve patient safety through a re-audit cycle. The audit standard was set as a ≤10% rejection rate, aligned with best practice recommendations from national hemovigilance guidance, serious hazards of transfusion (SHOT) and local transfusion policy.

## Materials and methods

Study design

This project was designed as a retrospective clinical audit conducted in accordance with the Standards for Quality Improvement Reporting Excellence (SQUIRE) framework. The audit reviewed G&S and crossmatch samples collected for pre-operative work-up of NOF patients to evaluate the frequency, causes, and cost implications of sample rejections occurring only in the A&E department.

Setting

The audit was carried out at the Luton & Dunstable Hospital, Bedfordshire NHS Foundation Trust, a district general hospital in the United Kingdom with a busy T&O service. The hospital's A&E department serves a high-volume population and performs routine preoperative blood grouping and antibody screening for trauma patients requiring surgical intervention. The G&S samples are usually collected in a Pink EDTA tube, and the patient's identification number, date of birth (DOB) and the name are labelled with a black ballpoint pen. The local transfusion policy statesthat the two samples must be collected 30 min apart and sent in two separate tubes, with the exact timing mentioned on each tube. The tubes are kept in separate covers with the printed requisition form, and the samples are then delivered to the laboratory for processing.

Study period

The initial audit covered a seven-month period, from September 2024 to March 2025. The potential solutions for improving the services were implemented in A&E in April 2025. A follow-up re-audit was conducted from 1st July 2025 to 1st October 2025.

Population selection criteria

All patients presenting with NOF fractures during the audit periods were included. These patients were selected because they routinely require urgent surgical management and preoperative blood typing.

Data collection

Data were obtained from the hospital's laboratory information management system (LIMS), A&E blood sample logs, and patient request forms. The unit of analysis was the G&S sample. Information collected included whether the G&S sample was accepted or rejected by the laboratory, the documented reason for rejection, the staff group who collected the sample, and the number of repeat samples required per patient.

Costing methodology

Financial cost estimates were derived from the hospital's local pathology finance reports (local pathology tariff). The direct cost per rejected sample was calculated by applying this tariff to the additional materials and processing required for a repeat test, summarised as a range (£12-£80) to reflect minor variations.

Data analysis

All collected data were entered into Microsoft Excel for descriptive statistical analysis. The primary denominator for calculating rejection rates was the total number of G&S samples processed during each audit period. Results were expressed as frequencies and percentages.

Classification of rejection categories

Rejection reasons documented by the laboratory were reviewed and grouped into three predefined categories: documentation errors (missing signature, missing date/time, mismatched patient identifiers between form and sample), sample quality issues (haemolysed, clotted, or underfilled samples), and labelling/ordering errors (duplicate requests, incorrect tube type used, addressograph label errors, ICE system reprint errors). The financial cost of each rejected sample was calculated based on local charges and summarised as a range per rejection event.

Ethical considerations

This project was undertaken as a clinical audit and quality improvement initiative; therefore, formal research ethics committee approval was not required. All data were anonymised, and patient identifiers were removed before analysis. The audit was conducted in compliance with institutional policies on confidentiality and data governance.

## Results

Initial audit findings (September 2024-March 2025)

**Figure 1:**
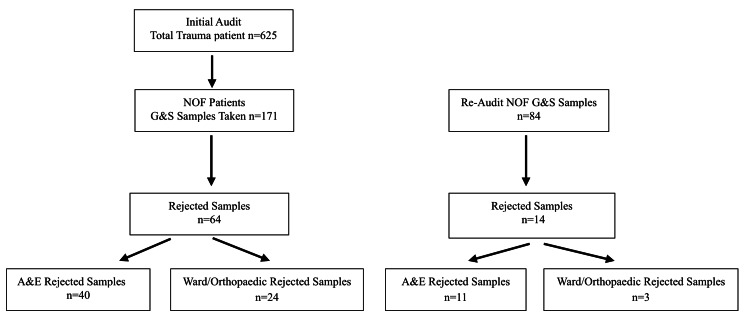
Flow Diagram of Patient Numbers and G&S Sample Rejections Across Initial Audit and Re-Audit Periods. n: number of patients or samples at each stage, A&E: Accident and Emergency department, Ward/Operating Theatre: samples collected on inpatient wards or in the operating theatre, Rejected samples: samples not accepted due to documentation errors, sample quality issues, or labelling/ordering errors, NOF: neck of femur fracture patients.

A total of 625 trauma patients were reviewed during the initial audit period from September 2024 to March 2025. From this cohort, 171 patients were admitted with NOF fractures and underwent preoperative G&S sampling. Among the 171 G&S samples collected, 64 were rejected, resulting in an overall rejection rate of 37.4%. The majority of these rejected samples (n=40) were collected in the A&E department, with the remaining 24 rejections occurring on the wards or in the operating theatre (Figure 1).

Repeated venepuncture was common among affected patients. Thirteen patients required repeat sampling two or more times due to initial rejections; one patient required G&S sampling three separate times before a valid result was obtained. This repetition directly contributed to patient discomfort and anxiety, as well as delays in preoperative workflow. In two cases (3.1%), rejections were attributed to internal laboratory errors rather than preanalytical issues.

The causes of sample rejection were grouped into three main categories: documentation errors (67.1%), sample quality issues (20.3%), and labelling and ordering errors (12.5%). The main reasons for rejections are summarised below (Tables 1, 2).

**Table 1:** Initial Audit Findings. Reasons for Group & Save sample rejections of neck of femur fractures. Percentages are of total rejections (n=64).

Category	Rejection causes	Frequency n (%)
Documentation errors	Missing signature, no date/time, mismatched identifiers	43 (67.1%)
Sample quality issues	Haemolyzed, clotted, or underfilled samples	13 (20.3%)
Labelling/order errors	Duplicate requests, wrong tube type, addressograph label, ICE reprint	8 (12.5%)
Total		64 (100 %)

**Table 2:** Re-Audit Findings. Reasons for Group & Save sample rejections of neck of femur fractures. Percentages are of total rejections in the re-audit period (n=14).

Category	Rejection causes	Frequency (%)
Documentation errors	Missing signature, no date/time, mismatched identifiers	8 (57.1%)
Sample quality issues	Haemolyzed, clotted, or underfilled samples	4 (28.6%)
Labelling/order errors	Duplicate requests, wrong tube type, addressograph label, ICE reprint	2 (14.3%)
Total		14 (100%)

## Discussion

This audit demonstrated that sample rejection in the A&E department represents a significant and preventable contributor to inefficiency within T&O patient care. Over 60% of rejected G&S samples in the initial cycle were collected in A&E, with most rejections attributed to documentation and labelling errors rather than analytical or laboratory-related faults. These findings are consistent with national hemovigilance reports, which indicate that identification and labelling discrepancies remain a predominant cause of pre-transfusion incidents [2,3,4]. Other specialities, such as general surgery, have found routine G&S testing unnecessary and not cost-effective for certain procedures [9]. This highlights the crucial role of the A&E department within the T&O patient flow pathway. 

The predominance of documentation-related errors underscores a critical gap in adherence to standard operating procedures (SOPs). Missing or incorrect patient identifiers, unsigned request forms, and absent timestamps were the most frequent causes of rejection. These issues often arise in the A&E environment, where time pressure, high patient turnover, and staff variability contribute to reduced compliance with documentation standards. While A&E staff aim to expedite care and efficiency and adhere to the 4-hour wait target, this can inadvertently increase preanalytical errors that delay subsequent surgical interventions, especially for NOF fracture patients who benefit most from early theatre scheduling [6,10].

Rejected samples not only delay laboratory processing and surgical clearance but also directly affect patient experience [6,9]. Repeated venepuncture increases patient discomfort and anxiety, while each rejection imposes additional workload on clinical and laboratory teams. Financially, the audit estimated an average direct cost of £30 per rejection based on local pathology finance reports, excluding hidden costs from staff time and delayed theatre use. This significant cost implication reiterates the value of high-quality quality improvement projects (QIPs) like this one and aligns with earlier studies highlighting the substantial economic burden associated with preanalytical errors in hospital laboratories [4,7,8].

While this audit did not directly measure downstream clinical outcomes such as time-to-theatre, adverse transfusion events, or patient satisfaction, the significant reduction in rejection rates indicates a substantial improvement in pre-operative workflow efficiency. By systematically addressing the root causes of sample rejection-primarily documentation and identification errors-the interventions implemented directly mitigate a known and significant source of pre-transfusion risk, as documented in national hemovigilance reports [3,4]. Therefore, the observed improvement strongly supports an enhancement in procedural safety for trauma patients undergoing urgent surgery.

The initial audit findings informed several targeted interventions: enhanced training and competency sessions for A&E staff, standardization of bedside labelling protocols, and the introduction of a novel, bedside G&S checklist developed by the audit team, which was affixed to all blood collection trolleys (Appendix A). These recommendations were presented and discussed at the A&E departmental audit meeting.

The success of these interventions was demonstrated in the re-audit, which showed a 20.8% reduction in the sample rejection rate (from 37.4% to 16.6%). This halving of the rejection rate following the introduction of the checklists is a key finding. The majority of rejections still appear to occur in the A&E department (40/64 and 11/14), necessitating the need for continued education, targeted training, refresher courses and regular re-audits. Most G&S sample rejections are preventable and can be effectively addressed through focused quality improvement measures that target human and procedural factors.

This audit was limited to a single institution and focused primarily on A&E samples for T&O patients. The data were descriptive and did not include detailed subgroup analysis of staff categories or patient outcomes. Nevertheless, the audit provides a clear baseline for targeted quality improvement and a successful model for future benchmarking against national standards.

The findings reaffirm that most G&S sample rejections are preventable and arise from modifiable human and procedural factors. By enhancing training, enforcing documentation standards, and integrating simple, practical safeguards, institutions can rapidly and significantly reduce rejection rates, save costs, and improve the timeliness of surgical care for trauma patients. 

Limitations of the study

The primary limitations of this study are its descriptive, single-centre design. While the audit cycle demonstrates a strong temporal association between the interventions and a reduced rejection rate, the observational nature of the data prevents definitive causal inference. The findings from one institution may not be directly generalisable to other settings with different workflows, staffing models, or patient populations.

Other important limitations should be acknowledged. First, a potential Hawthorne effect may have followed the staff education and introduction of the checklist, temporarily improving compliance. Second, changes in staff rotation, experience levels, or seasonal variations in A&E workload between the audit and re-audit periods could have influenced error rates independently of the interventions. Third, this audit did not directly measure several important downstream outcomes, such as specific delays to theatre time, adverse transfusion events, time-to-transfusion, or patient satisfaction, which limits the strength of claims regarding direct clinical impact on patient safety. Finally, the cost analysis considered direct pathology tariffs but did not perform a comprehensive analysis of indirect costs such as nursing time or theatre delays.

Therefore, to strengthen the evidence, future work should involve controlled, multi-centre studies to assess the generalisability and definitive impact of such interventions and incorporate direct measurement of clinical and operational outcomes.

## Conclusions

This clinical audit identified documentation and labelling deficiencies as the predominant causes of G&S sample rejection in the A&E department. These avoidable preanalytical errors accounted for most of the delays in perioperative management of T&O patients, particularly those with NOF fractures requiring urgent surgery. This finding is particularly important as feedback to improve the service, as this issue has been flagged up multiple times.

The implementation of standardised bedside labelling protocols, targeted staff training, and the introduction of a G&S checklist led to a marked improvement, as evidenced by the re-audit, which showed a marked reduction in the rejection rate. This is the key take-home message from the study. Continuous monitoring through repeat audit cycles is recommended to ensure sustained improvement and to evaluate the long-term impact of these interventions on patient safety, workflow efficiency, and resource utilisation. The successful outcome of this quality improvement project underscores the value of a systematic approach to addressing preanalytical errors in a high-pressure clinical environment.

## Figures and Tables

**Figure 2:**
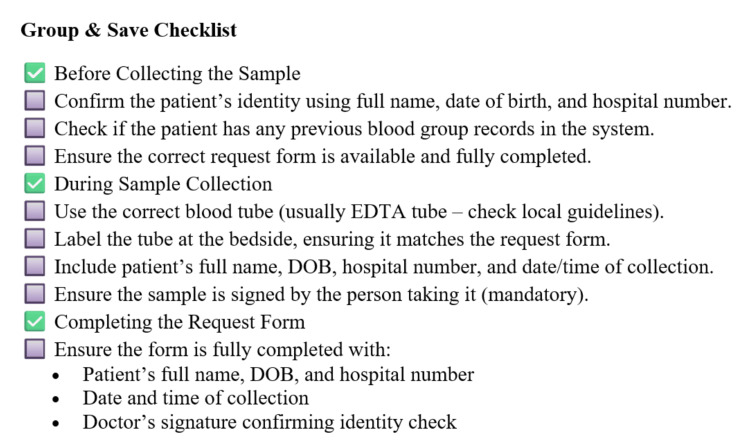
Locally Developed Checklist Used for Group and Save (G&S) Sample Collection and Processing Novel Group & Save checklist implemented during the intervention period. Designed by the audit team, this checklist outlines the critical steps for patient identification, sample collection, tube labelling, and form completion to mitigate common documentation and labelling errors identified in the initial audit.

## Appendices

Appendix A 
